# Large-scale analysis of protein crotonylation reveals its diverse functions in *Pinellia ternata*

**DOI:** 10.1186/s12870-022-03835-y

**Published:** 2022-09-23

**Authors:** Weiwei Guo, Jiayi Han, Ximei Li, Zihan He, Yumei Zhang

**Affiliations:** Shandong Provincial Key Laboratory of Dry Farming Technology/Shandong Engineering Research Center of Germplasm Innovation and Utilization of Salt-Tolerant Crops/College of Agronomy, Qingdao Agricultural University, Qingdao Shandong, 266109 China

**Keywords:** Posttranslational modification, Lysine crotonylation, Crotonylome, *Pinellia ternata*

## Abstract

**Background:**

*Pinellia ternata* is an important traditional medicine in China, and its growth is regulated by the transcriptome or proteome. Lysine crotonylation, a newly identified and important type of posttranslational modification, plays a key role in many aspects of cell metabolism. However, little is known about its functions in *Pinellia ternata*.

**Results:**

In this study, we generated a global crotonylome analysis of *Pinellia ternata* and examined its overlap with lysine succinylation. A total of 2106 crotonylated sites matched on 1006 proteins overlapping in three independent tests were identified, and we found three specific amino acids surrounding crotonylation sites in *Pinellia ternata*: K^cr^F, K***Y**K^cr^ and K^cr^****R. Gene Ontology (GO) and KEGG pathway enrichment analyses showed that two crucial alkaloid biosynthesis-related enzymes and many stress-related proteins were also highly crotonylated. Furthermore, several enzymes participating in carbohydrate metabolism pathways were found to exhibit both lysine crotonylation and succinylation modifications.

**Conclusions:**

These results indicate that lysine crotonylation performs important functions in many biological processes in *Pinellia ternata*, especially in the biosynthesis of alkaloids, and some metabolic pathways are simultaneously regulated by lysine crotonylation and succinylation.

**Supplementary Information:**

The online version contains supplementary material available at 10.1186/s12870-022-03835-y.

## Background

Posttranslational modifications (PTMs), e.g. the introduction of novel functional groups on lysine residues, such as acetyl, phosphoryl, ubiquityl, succinyl, crotonyl and methyl groups [1], often occur during or after protein biosynthesis, and are involved in the regulation of diverse cellular processes. Among these modifications, the crotonylation of lysine is one of the recently discovered acyl modifications.

Lysine crotonylation (Kcr) was first identified on histone proteins and mainly occurs at the ε-amino group of lysine, which is a chemical modification similar to acetylation [2, 3]. Previous evidence indicated that lysine crotonylation and acetylation sites overlapped in histones, but crotonylation sites were not redundant with acetylation sites [4]. The balance of lysine crotonylation is regulated by protein crotonyl-transferases and decrotonylases [5]. Histone crotonylation is recognized as an indicator of active genes, plays an important role in the regulation of global transcription in mammalian cells, and participates in the male germ cell differentiation process [3, 6]. Recently, more nonnuclear proteins were discovered to be crotonylated [7, 8], and the crotonylation of nonhistone proteins was shown to be involved in the regulation of signalling transduction, the cell cycle, cellular metabolism and cellular processes [9–11].

*Pinellia ternata* (Thunb.) Beri is a perennial herb belonging to Araceae. It has been widely utilized as an important component of traditional Chinese medicine for thousands of years. The tuber of *P. ternata* exhibits specific pharmacological properties, such as antiemetic, expectorant, antipyretic and styptic effects [12–17]. Although the demand for *P. ternata* is increasing in China, sources are becoming scarce because of overexploitation. With the development of bioinformatics, molecular biology, and proteomics, large amounts of omics analysis have been performed on *P. ternata*. The transcriptome of *P. ternata* under normal and shaded environments provided a foundation for further study of this major traditional herb [18]. Zhu et al. (2013) detected 27 heat-responsive proteins by using two-dimensional electrophoresis [19].

To date, no global analysis of lysine crotonylation in *P. ternata* has been reported. In this study, we performed the first proteome-wide analysis of lysine crotonylation in the leaves of *P. ternata* with the purpose of providing more information about metabolic regulation in the biosynthesis of ephedrine alkaloids. This study may offer insights into the functional characterization of lysine crotonylation in *P. ternata*.

## Results

### Proteome-wide analysis of lysine crotonylation in leaves of *P. ternata*

**Fig. 1:**
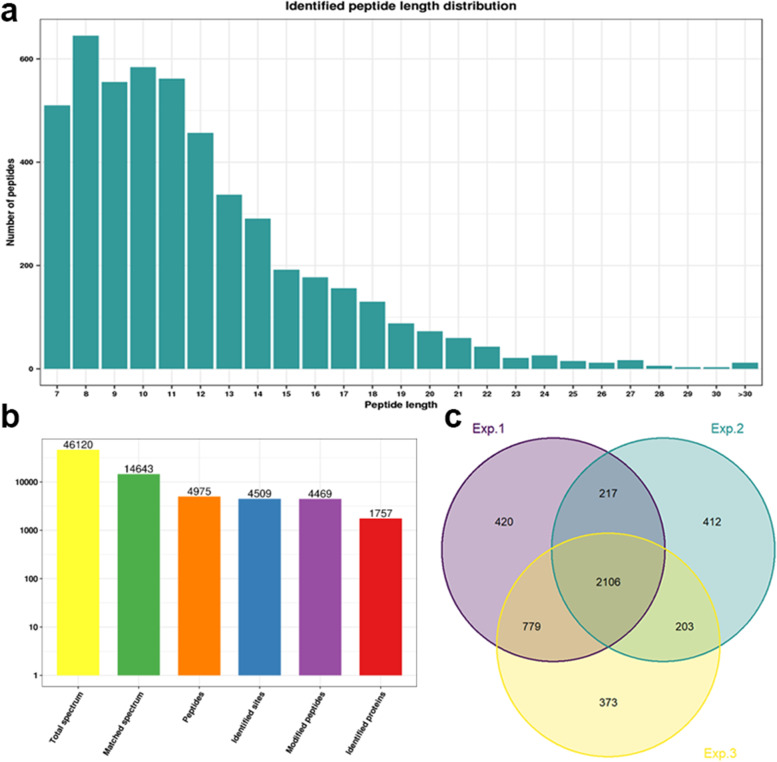
Distribution of crotonylated peptides based on their length (**a**), Basic statistical analysis of MS results (**b**) and Venn diagram of modification sites identified in replicates (**c**). The analysis was performed based on 2106 crotonylated sites matched on 1006 overlapping proteins in three independent tests

Lysine crotonylation, a newly discovered PTM, occurs in both prokaryotes and eukaryotes [20–25]. However, until now, it has rarely been studied in *P. ternata*. To investigate the changes in lysine crotonylation sites, the proteins of *P. ternata* leaves were extracted and digested into peptides by trypsin. Consistent with the properties of tryptic peptides, the length of most peptides ranged from 7 to 20 amino acids (Fig. 1a). To obtain accurate data, three independent biological replications were performed, and a total of 1494, 1292 and 1470 crotonylated proteins were separately identified. In total, 4509 crotonylated sites on 1757 proteins were found, among which 2106 crotonylated sites in 1006 proteins were identified simultaneously in all three biological replicates (Fig. 1b and c, Supplementary Table S1). Notably, 55.6% of the identified proteins had only one crotonylated lysine site, and 20.9% of the identified proteins had two crotonylated lysine sites (Fig. S1).

### Motif analysis of lysine crotonylation sites

**Fig. 2:**
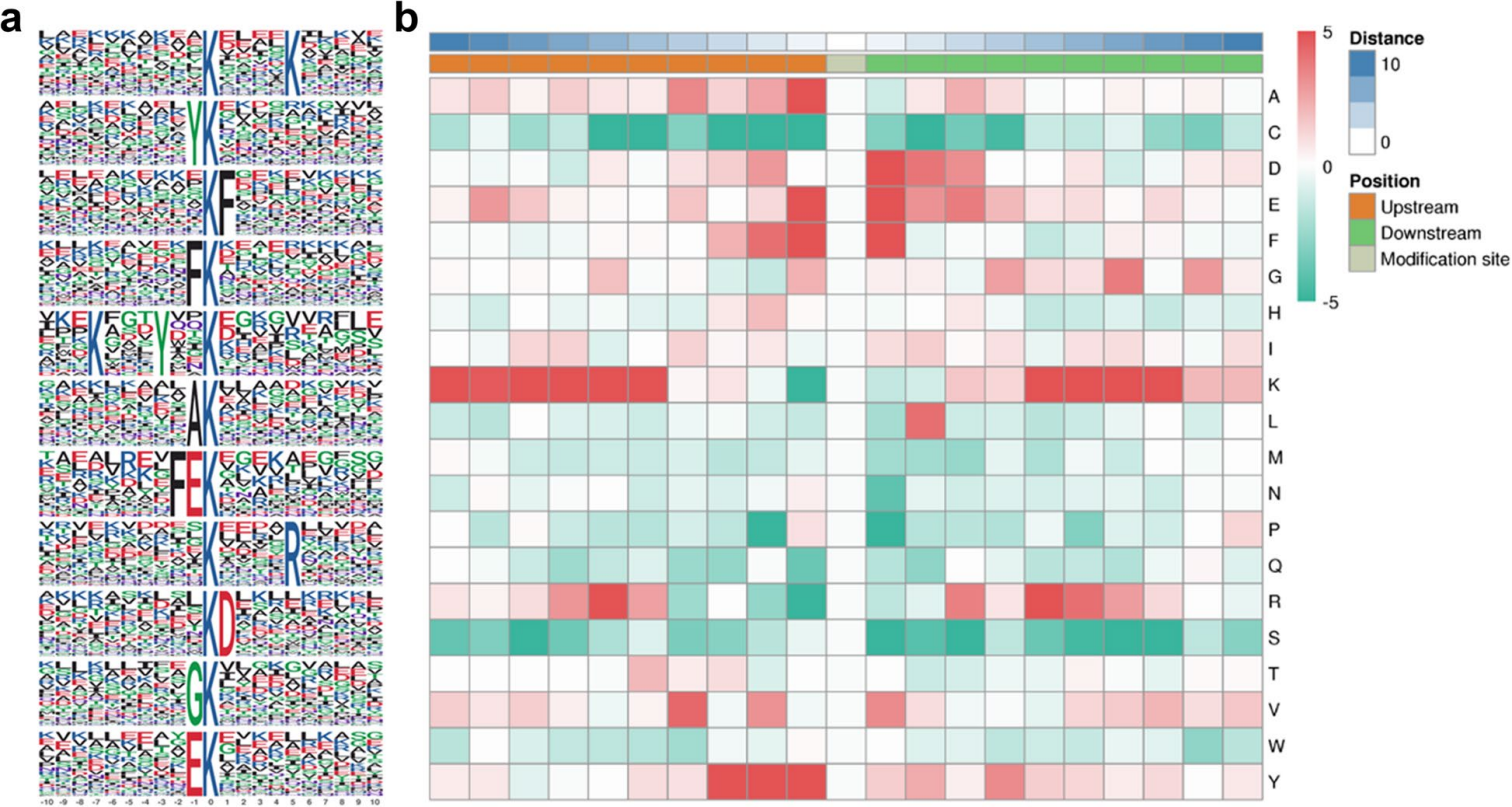
Properties of lysine-crotonylated peptides. **a** Crotonylation sequence motifs for ± 10 amino acids around the lysine crotonylation sites. **b** Heatmap of the amino acid compositions of the crotonylated sites. The analysis was performed based on 2106 crotonylated sites matched on 1006 proteins overlapping in three independent tests

To evaluate the properties of crotonylation sites in *P. ternata* leaves, sequence motif analysis was performed using the Motif-x program. Eleven conserved motifs were enriched surrounding the crotonylated lysine site with amino acid sequences from the − 10 to + 10 position, including K^cr^****K, YK^cr^, K^cr^F, FK^cr^, K***Y**K^cr^, AK^cr^, FEK^cr^, K^cr^****R, K^cr^D, GK^cr^ and EK^cr^ (K^cr^ indicates the lysine crotonylation site and * represents a random amino acid residue) (Fig. 2a). All the motifs displayed different abundances, such as K***Y**K^cr^, which was the most enriched, followed by FEK^cr^ (Fig. 2a, Supplementary Table S1). Comparing these consensus motifs between *P. ternata* and other plants showed that many conserved motifs including K^cr^D and EK^cr^, are shared in rice, tea and tobacco [26–28]. It is worth noting that K^cr^F, K***Y**K^cr^ and K^cr^****R were specific to *P. ternata*, indicating that these three motifs are important in this medicinal herb.

To analyse the enrichment of amino acid residues around the K^cr^ sites, a heatmap was generated. In accordance with the conserved motifs K^cr^F and FK^cr^, phenylalanine (F) was enriched in the + 1 and -1 positions near crotonylation sites, suggesting that it is a widespread amino acid around the crotonylated sites (Fig. 2b). Additionally, aspartic acid (D) was enriched in the + 1 position but deleted downstream, and tyrosine (Y) and alanine (A) were significantly overrepresented at the -1 position. Remarkably, the frequency of lysine (K) was highly represented in the + 5 and -5 positions (Fig. 2b).

### Secondary structure analysis of crotonylated proteins

**Fig. 3:**
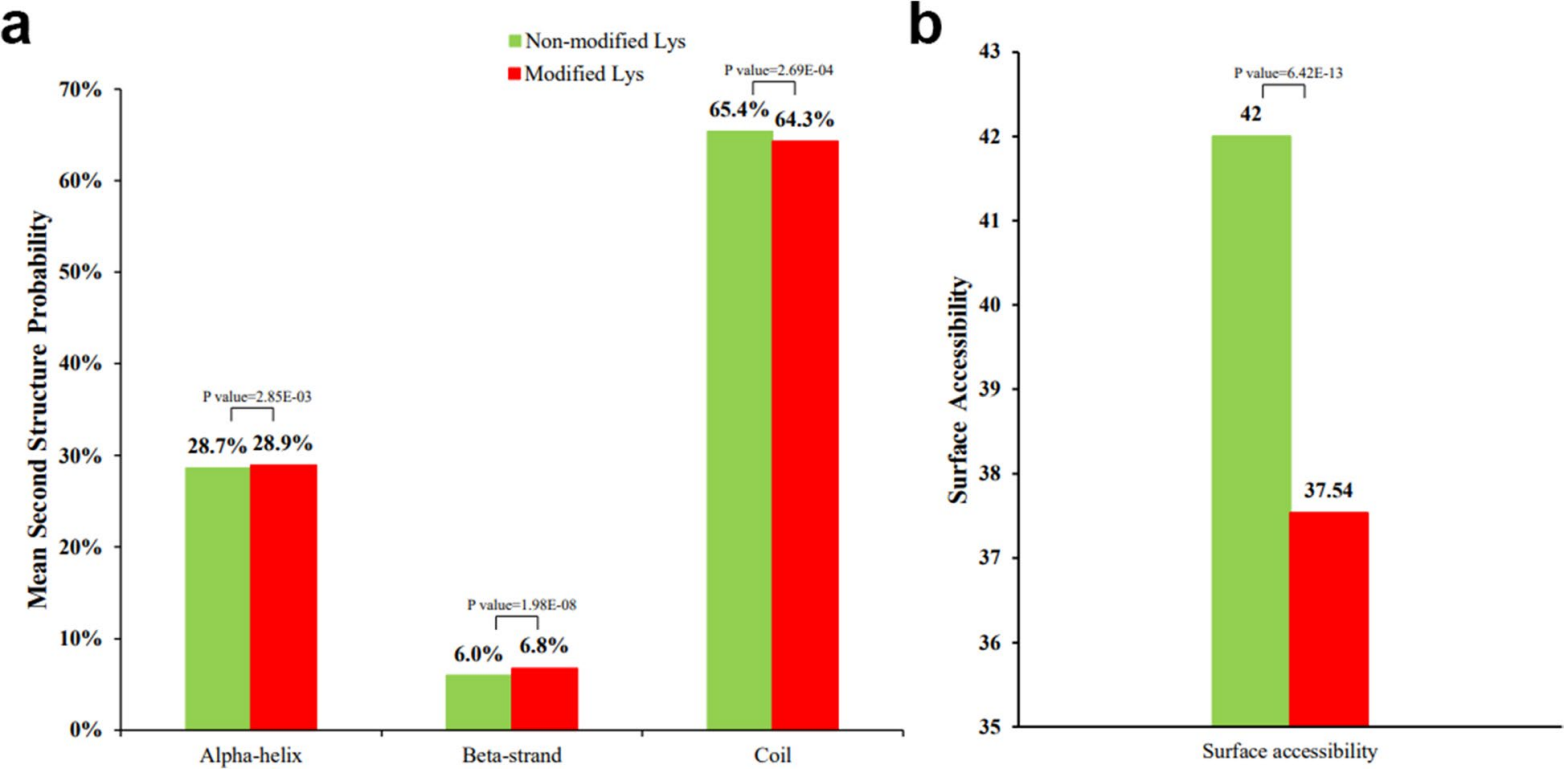
Probabilities of lysine crotonylation in different protein secondary structures (alpha-helix, beta-strand and coli) (**a**) and predicted surface accessibility of acetylation sites (**b**). All lysine sites are shown in green, and crotonylated lysine sites are shown in red. The analysis was performed based on 2106 crotonylated sites matched on 1006 proteins overlapping in three independent tests

To analyse the relationship between protein structure and lysine crotonylation in *P. ternata*, a secondary structure analysis was conducted. A total of 35.7% of crotonylated sites were located in ordered regions, and 28.9% and 6.8% of sites were located in alpha-helix and beta-strand regions, respectively (Fig. 3a). Further analysis of surface accessibility demonstrated that 37.54% of lysine crotonylation sites were exposed to the protein surface, indicating that the properties of surface proteins are not easily affected by lysine crotonylation (Fig. 3b).

### Functional annotation and cellular localization of crotonylated proteins in *P. ternata*

**Fig. 4:**
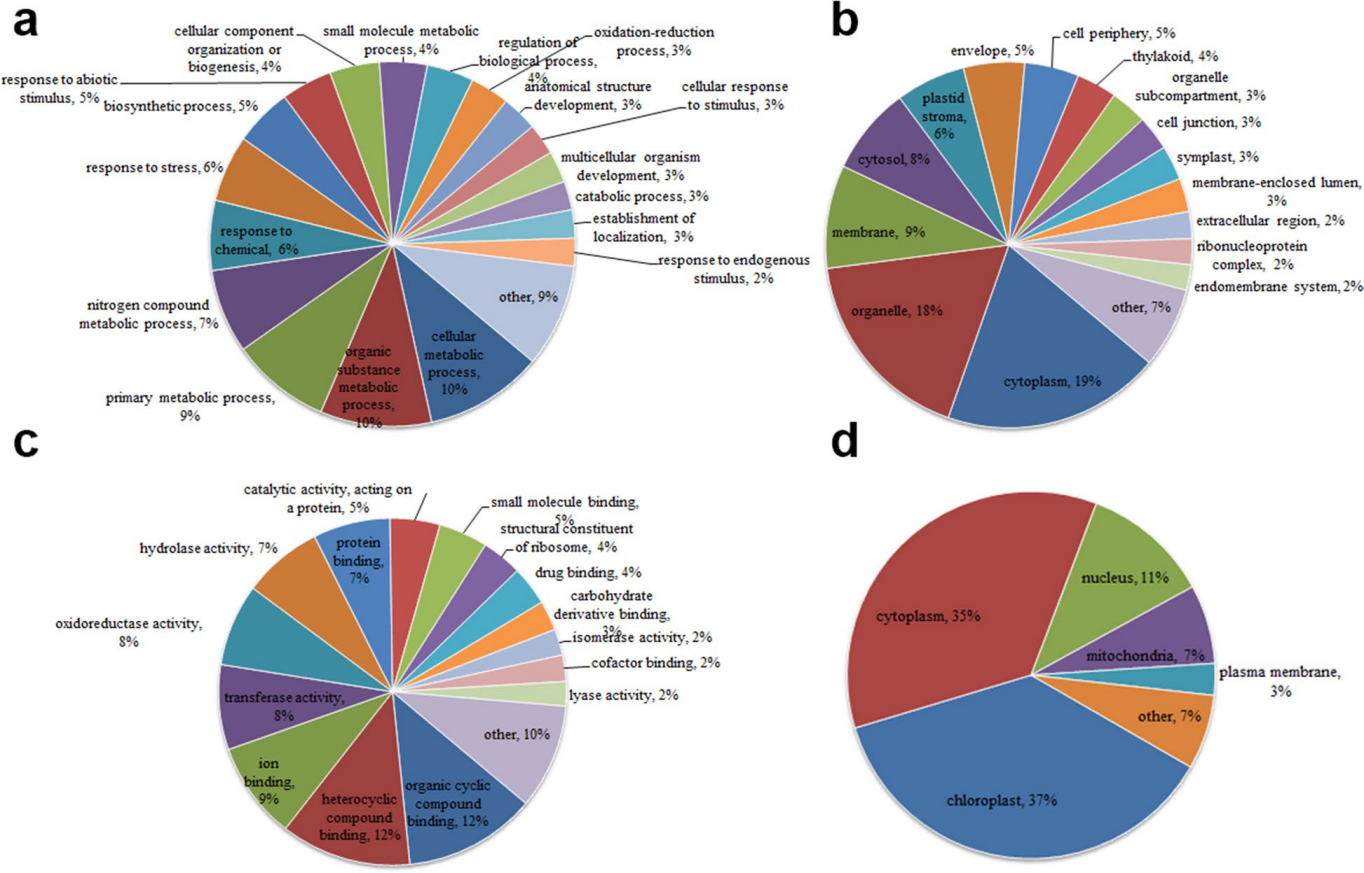
Functional classification of acetylated proteins in *P. ternata*. **a** Classification of the crotonylated proteins based on biological process, **b** classification of the crotonylated proteins based on cellular component, **c** classification of the crotonylated proteins based on molecular function, **d** subcellular localization of the crotonylated proteins. The analysis was performed based on 2106 crotonylated sites matched on 1006 proteins overlapping in three independent tests

To elucidate the roles of lysine crotonylation in *P. ternata*, a Gene Ontology (GO) functional classification analysis was performed (Fig. 4, Supplementary Table S2). In the biological process category, more crotonylated proteins were enriched in cellular metabolic processes (10%), organic substance metabolic processes (10%), and primary metabolic processes (9%) (Fig. 4a). The cellular component analysis showed that most modified proteins were distributed in the cytoplasm (19%) and organelles (18%) (Fig. 4b). Consistent with these results, a number of crotonylated proteins were found to be related to protein binding and enzyme activities in the molecular function classification (Fig. 4c).

Subcellular localization prediction analysis was conducted with WolfPsort software, and the results showed that most crotonylated proteins were distributed in the chloroplast (37.01%) and cytoplasm (35.42%). Meanwhile, some proteins were predicted to be distributed in the nucleus (11.24%), mitochondria (6.97%), and plasma membrane (2.79%) (Fig. 4d). Taken together, these data indicate that lysine crotonylated proteins play diversified roles in biological processes in *P. ternata*.

### Enrichment-based cluster analysis of crotonylated proteins in *P. ternata*

**Fig. 5:**
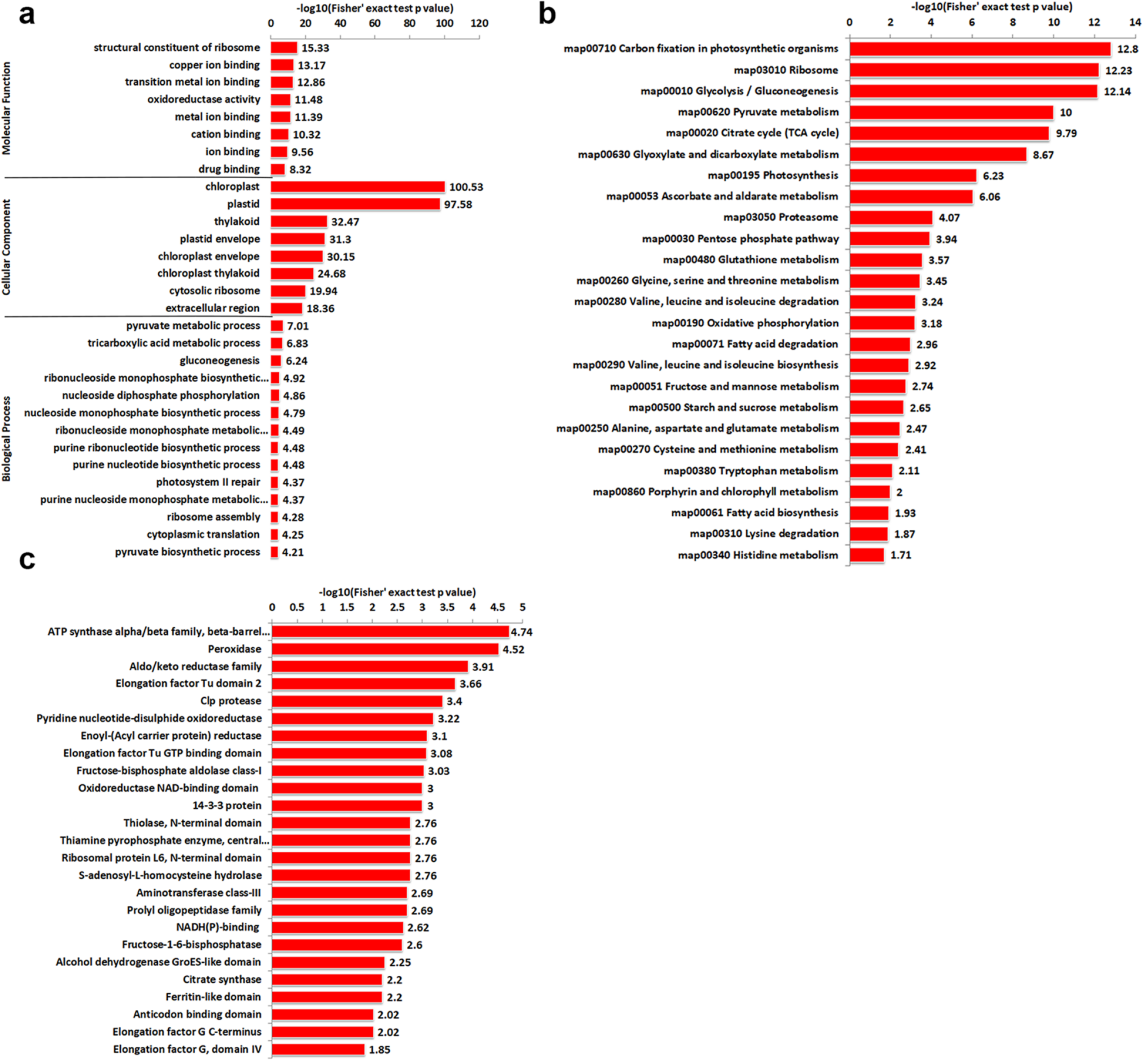
Functional enrichment based cluster analysis of crotonylated proteins in *P. ternata*. **a** GO-based enrichment analysis of lysine crotonylation, **b** KEGG pathway analysis of lysine crotonylation, **c** protein domain analysis of lysine crotonylation. The analysis was performed based on 2106 crotonylated sites matched on 1006 proteins overlapping in three independent tests

To further understand the function of lysine crotonylation in *P. ternata*, a GO enrichment analysis based on molecular function, cellular component and biological process was performed (Fig. 5a, Supplementary Table S3). According to the molecular function enrichment results, proteins related to structural constituent of ribosome, copper ion binding, transition metal ion binding, oxidoreductase activity, metal ion binding and cation binding were greatly enriched (Fig. 5a). Regarding cellular component enrichment analysis among crotonylated sites, chloroplast- and plastid- related categories were more enriched (Fig. 5a). Furthermore, in the analysis of biological process enrichment, the pyruvate metabolic process, tricarboxylic acid metabolic process and gluconeogenesis were more enriched for crotonylated proteins (Fig. 5a).

KEGG pathway enrichment analysis was implemented to study the role of these crotonylated proteins in *P. ternata* (Fig. 5b, Supplementary Table S4). The results indicated that carbon fixation in photosynthetic organisms, ribosome, and glycolysis/gluconeogenesis pathways were more enriched in the leaves of *P. ternata*. Similar results were also observed in the protein domain enrichment analysis, and ATP synthase alpha/beta family and beta-barrel domain proteins were also greatly enriched (Fig. 5c, Supplementary Table S5).

Taken together, lysine crotonylated proteins participated in a wide variety of pathways, indicating an important role of the new posttranslation modification in *P. ternata*.

### Crotonylation of proteins involved in multiple biological processes

Photosynthesis, one of the major metabolic processes, converts light to energy [21]. In our study, a total of 43 key enzymes were crotonylated (Supplementary Table S6). Among them, one subunit of photosystem I (PsaF) and five subunits of photosystem II (Psb28, PsbC, PsbQ, Psb27 and PsbP) were crotonylated (Supplementary Table S6). Additionally, fructose-1,6-bisphosphatase (FBPase), ribulose-1,5-bisphosphate carboxylase/oxygenase (Rubisco) and phosphoenolpyruvate carboxylase (PEPC) were also crotonylated and were reported to be the core enzymes in carbon fixation pathways [29–31] (Supplementary Table S6).

Notably, we found 153 crotonylated sites in 45 stress-related proteins with crotonylation changes (Supplementary Table S6). Many well-known abiotic stress-responsive proteins, such as 70-kD and 90-kD heat shock proteins (HSPA1, HSPA4, HSPA9 and HSP90), 14–3-3 protein, BAG family, ABC transporter C family and late embryogenesis abundant (LEA) proteins, were all modified by crotonyl groups (Supplementary Table S6).

In the biosynthesis of the ephedrine pathway, two key enzymes were crotonylated. ThDP-dependent pyruvate decarboxylase (PDC) and acetolactate synthase (AHAS) had one and two crotonylation sites, respectively (Supplementary Table S6). These results indicated that lysine crotonylation may participate in the regulation of the ephedrine content in *P. ternata*.

Phytohormones, such as auxin and abscisic acid (ABA), are necessary for plant development [32]. In our study, some hormone signalling component proteins were crotonylated, such as three auxin-related proteins (PIN1, SNX1 and VPS) and one ABA-related protein (PP2C) (Supplementary Table S6).

### Overlapping analysis between lysine crotonylation and succinylation in leaves of *P. ternata*

**Fig. 6:**
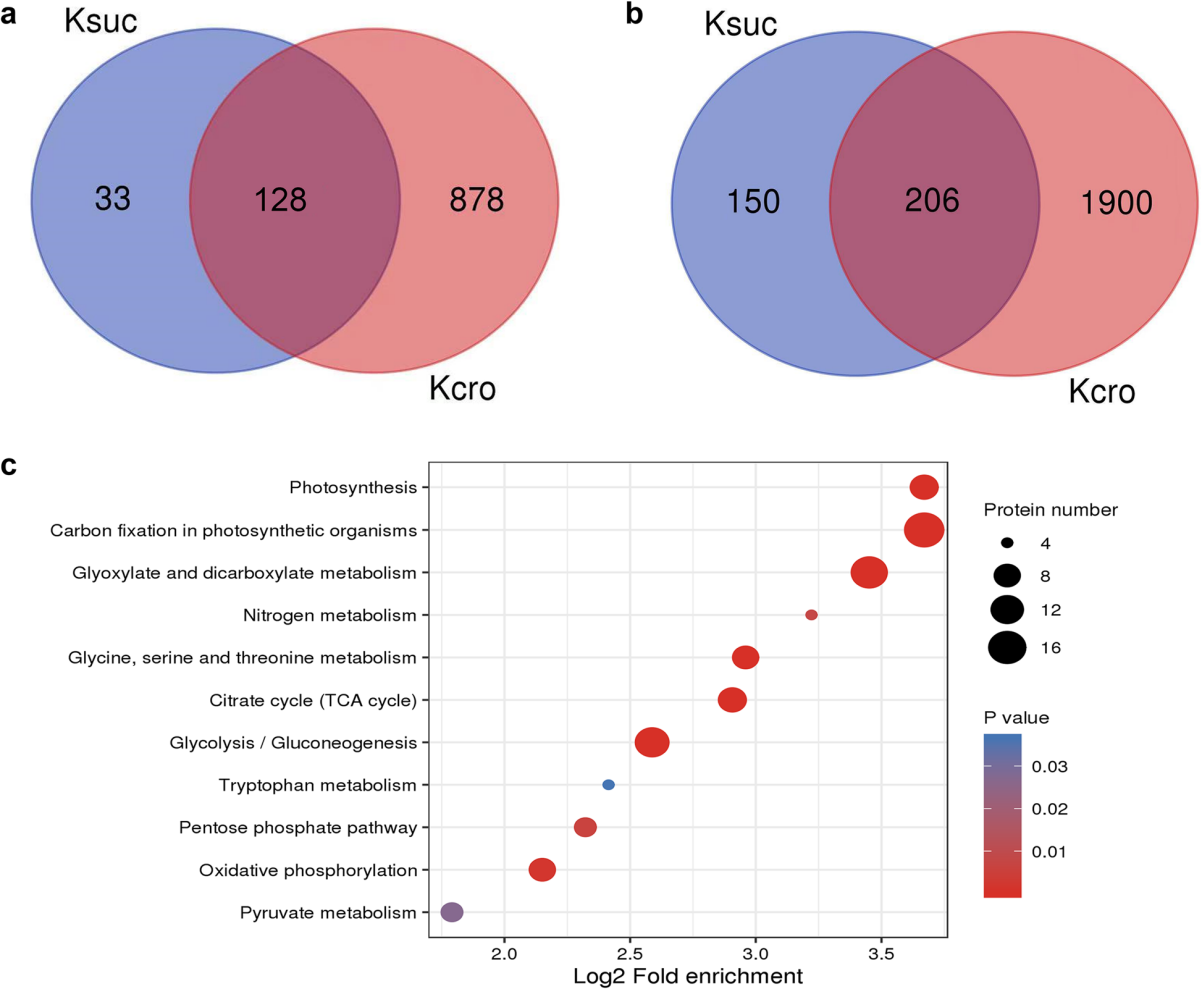
Overlap between lysine crotonylation and succinylation in *P. ternata*. **a** Overlap of crotonylated proteins and succinylated proteins, **b** overlap of crotonylated proteins and succinylated sites, **c** overlap of crotonylated proteins and succinylated proteins based on KEGG pathway enrichment analysis. The analysis was performed based on 2106 crotonylated sites matched on 1006 proteins overlapping in three independent tests

Lysine succinylation, which participates in a variety of crucial biological processes, is another important PTM [33, 34]. In this study, we performed succinylome analysis of *P. ternata*, and 356 sites in 161 proteins were found to be succinylated. To elucidate the relationship between crotonylation and succinylation of the same lysine residue, the lysine crotonylome and the succinylome were both analysed. A total of 128 proteins and 206 sites were modified by both crotonylation and succinylation (Fig. 6a and b). Furthermore, 13 crotonylated sites of 8 HSPs were also succinylated. Moreover, some enzymes in carbohydrate metabolism were also found to be both crotonylated and succinylated, such as glyceraldehyde-3-phosphate dehydrogenase, pyruvate dehydrogenase, and fructose-bisphosphate aldolase. KEGG pathway analysis showed that proteins related to the oxidative phosphorylation pathway, pentose phosphate pathway, glycolysis/gluconeogenesis pathway, TCA cycle pathway, glycine, serine and threonine metabolism pathway, glyoxylate and dicarboxylate metabolism pathway, carbon fixation in photosynthetic organisms pathway, and photosynthesis pathway were both crotonylated and succinylated (Fig. 6c). Among these pathways, carbon fixation in photosynthetic organisms was found to be the most enriched. These results indicate that crotonylation and succinylation can coordinately regulate many important biological processes in *P. ternata*.

## Discussion

### Lysine crotonylation plays an important role in photosynthesis and carbon fixation processes in *P. ternata*

Plenty of evidence has indicated that PTMs participate in many metabolic pathways, such as photosynthesis, carbon fixation, physiological regulation, and stress response [2, 23]. Lysine crotonylation, one of the most important PTMs, was first found in histone proteins. Recently, an increasing number of studies have focused on the crotonylation modification of nonhistone proteins in plants, such as rice, peanut, chrysanthemum, and tobacco [35–38]. However, it has been barely examined in *P. ternata*. In this study, we performed global crotonylome analysis of *P. ternata*. A total of 1006 crotonylated proteins were identified in the leaves, which provides an opportunity to investigate the function of lysine crotonylation (Fig. 1b, Supplementary Table S1). Although the number may be underestimated because of technical issues and the inherently dynamic nature of lysine crotonylation in *P. ternata*, many important proteins were found to be crotonylated. For example, six proteins participating in photosynthesis, including one subunit of photosystem I (PsaF) and five subunits of photosystem II (Psb28, PsbC, PsbQ, Psb27 and PsbP), were found to be crotonylated (Supplementary Table S6). These proteins were also reported to be regulated by lysine crotonylation in rice [35]. Consistent with the expected results, crotonylated proteins were enriched in photosynthetic pathways in the leaves of *P. ternata*.

Carbon fixation can consume energy (such as ATP, GTP and NADPH) to form glucose, which also acts as the product of photosynthesis. There are many key enzymes involved in carbon fixation, such as ribulose-1,5-bisphosphate carboxylase/oxygenase (Rubisco), fructose-1,6-bisphosphatase (FBPase) and phosphoenolpyruvate carboxylase (PEPC) [29–31]. Previous studies have found that reducing the expression of Rubisco could result in lower rates of carbon assimilation in tobacco [29], and reducing FBPase expression could weaken photosynthesis and plant growth in potato [30]. The data in our study showed that the lysine residues of all three enzymes were extensively crotonylated in the leaves of *P. ternata* (Supplementary Table S6). Overall, these data suggested that lysine crotonylation might play an important role in photosynthesis and carbon metabolism in *P. ternata*.

### Lysine crotonylation analysis of stress-related proteins

Due to changes in the external environment, plants have evolved numerous mechanisms to deal with intricate stimuli. Some proteins have been widely studied and proven to be important for plant adaptation, for example, heat-shock proteins (HSPs), which are crucial chaperonins [24, 39]. HSP70 and HSP90 were reported to be subjected to lysine crotonylation in tobacco, and their orthologues were also crotonylated in the leaves of *P. ternata* (Supplementary Table S6) [38]. In addition, 14–3-3 protein, another important abiotic stress-responsive protein, was reported to play pivotal roles in many signal transduction cascades [40] and was also found to be crotonylated in the present study (Supplementary Table S6). Moreover, some other crucial stress-related proteins, such as the BAG family, ABC transporter C family, and LEA proteins, were all identified as crotonylated in this study (Supplementary Table S6). We speculate that Kcr might contribute to the stress-related pathway during the development of leaves in *P. ternata*. Due to the lack of treatment experiments, the regulatory mechanism needs to be further studied. Nevertheless, this global characterization of lysine crotonylation will improve our understanding of the relationship between Kcr and stress-related proteins in the leaves of *P. ternata*.

### Lysine crotonylation contributes to the biosynthesis of ephedrine alkaloid in *P. ternata*

Alkaloids are one of the most important medicinal compounds in *P. ternata*, among which ephedrine is the main ingredient that belongs to phenylpropylamino alkaloids. Studies have shown that ephedrine plays an important role in clinical treatment, such as bronchial asthma, nasal mucosal congestion and nasal congestion [41, 42]. Therefore, it is desirable to improve the quality of *P. ternata* by increasing the content of ephedrine.

The biosynthesis of ephedrine comprises complex branching biochemical pathways (Supplementary Table S6). In the first step, L-Phe ammonia-lyase (PAL) catalyses the deamination of L-Phe to trans-cinnamic acid (CA). Crue (1988) found that benzoic acid is an intermediate in the synthesis of amphetamines, which are synthesized through non-β-oxidative CoA-independent pathways [43]. ThDP-dependent pyruvate decarboxylase (PDC) or acetolactate synthase (AHAS) can catalyse the conversion of benzoic acid to 1-phenylpropane-1,2-dione [44]. Here, we found that PDC and AHAS were both crotonylated. Nevertheless, the role of lysine crotonylation in the regulation of ephedrine biosynthesis in *P. ternata* requires further investigation.

### Analysis of hormone signalling pathways involved in the development of *P. ternata*

Plant hormones, mainly auxin, abscisic acid (ABA), gibberellic acid (GA), ethylene, cytokinin (CTK), and brassinosteroids (BRs), play vital roles in the regulation of plant growth and development [32]. Many studies have found that auxin participates in diverse developmental and physiological processes [45, 46]. PIN1, a polar transport protein, plays an important role in the polar transport of auxin [47]. In this study, we found that PIN1 could be crotonylated. Moreover, SNX1 (SORTING NEXIN 1), which has been reported to regulate PIN2 degradation or recycling [48, 49], was also crotonylated (Supplementary Table S6). Furthermore, vacuolar protein sorting (VPS) proteins, another crucial component of the plant retromer complex, were highly crotonylated in the leaves of *P. ternata* (Supplementary Table S6).

ABA, another important phytohormone, functions in many aspects of plant development, such as seed germination, dormancy, seeding growth and seed maturation [50]. A number of components were found to participate in the ABA signalling pathway. For example, type-2C protein phosphatase (PP2C) was reported to turn ABA signalling off through interaction with SNF1-related protein kinases (SnRKs) [51–53]. In our study, PP2C protein was found to be crotonylated (Supplementary Table S6). Similar results were also found in other plants, such as papaya [2]. However, whether and how lysine crotonylation participates in the phytohormone signalling pathway in *P. ternata* remains to be elucidated.

### Lysine crotonylation plays an important role during the development of *P. ternata* and functions similarly to lysine succinylation

Recently, an increasing number of lysine modifications of nonhistone proteins have been identified. As major PTMs, protein lysine crotonylation and succinylation have been detected in many plants, such as wheat, rice, peanut, and tobacco [20, 35, 36, 38]. However, they are barely known in *P. ternata*. Our data showed that the proteins related to the oxidative phosphorylation pathway, pentose phosphate pathway, glycolysis/gluconeogenesis pathway, TCA cycle pathway, glycine, serine and threonine metabolism pathway, glyoxylate and dicarboxylate metabolism pathway, carbon fixation in photosynthetic organisms pathway, and photosynthesis pathway displayed an increased tendency to be both crotonylated and succinylated in the KEGG pathway analysis (Fig. 6c). In accordance with these results, several crucial enzymes of carbohydrate metabolism were also modified simultaneously by lysine crotonylation and succinylation. Interestingly, 13 crotonylated sites of 8 heat-shock proteins (HSPs), well-known stress-related proteins, were also found to be succinylated. This phenomenon indicates that lysine crotonylation and succinylation have some common connections in *P. ternata*.

## Conclusions

In summary, 2106 overlapping crotonylated sites on 1006 proteins, belonging to various functional families, were identified in the leaves of *P. ternata*. These results indicate that lysine crotonylation might participate in various biological processes. Moreover, our results revealed that K^cr^F, K***Y**K^cr^ and K^cr^****R were the specific sites of this PTM in *P. ternata*. Two key alkaloid biosynthesis related enzymes, which are important medicinal compounds, were found to be crotonylated in this study. These results suggest that lysine crotonylation may play an important role in the medication treatment of *P. ternata*. Stress-related proteins were also found to have crotonylation changes, and several enzymes involved in carbohydrate metabolism could be both crotonylated and succinylated. Taken together, these data provide new insights into the molecular mechanisms of lysine crotonylation in the leaves of *P. ternata*. In the present study, leaves of three-week-old plants were chosen for analysis, and whether some other tissue-specific proteins are modified by lysine crotonylation still needs to be investigated.

## Materials and methods

### Plant material and growth conditions

Thetubers of *Pinellia ternata* (Thunb.) used in this study were collected from Gansu, China (34.0°N latitude and 105.3°E longitude). The plant specimen was deposited in the Chinese Virtual Herbarium with an herbarium ID of WUK 5,971,434 (http://ppbc.iplant.cn/), and the tubers were identified by Renbin Zhu. The tubers were planted in nutrient soil in a greenhouse with a relative humidity of 50% and a temperature of 22 °C during the day and 18 °C at night. The leaves of three-week-old seedlings were collected, snap-frozen in liquid nitrogen and stored at -80 °C for crotonylome analysis.

### Protein extraction and trypsin digestion

Proteins from *Pinellia ternata* leaves were extracted as described in previous studies [19, 20, 54, 55]. Approximately 0.4 g of fresh leaf material was ground in liquid nitrogen and sonicated three times in lysis buffer, and then the remaining debris was depleted by centrifugation at 20,000 × g for 10 min at 4 °C [21]. The supernatant was precipitated by trichloroacetic acid (TCA) at -20 °C for at least 2 h and then the protein was collected by centrifugation [21]. After alkylation and dilution, a two-step trypsin digestion was carried out according to Zhou et al. (2016) [54].

### HPLC fractionation and affinity enrichment

After trypsin digestion, the peptides were fractionated into 80 fractions by high pH reverse-phase HPLC using Ultimate RSLCnano 3000. For affinity enrichment, the fractions of peptide were incubated with pan anti-crotony lysine antibody beads [20]. The lysine crotonylated peptides bound to the agarose beads were eluted with 0.1% trifluoroacetic acid, after being washed four times with NETN buffer and twice with double distilled water and the eluted fractions were vacuum-dried for further use [20].

### LC–MS/MS analysis

The enriched crotonylated peptides were investigated by mass spectrometry as described previously [20–22, 54, 55]. The protein integrity was detected by the Orbitrap at a resolution of 70,000 (m/z 200) with an NCE setting of 30. The m/z range was set from 350 to 1800 for the MS scan [20–22, 54, 55]. The LC–MS/MS analysis was performed blindly by Micrometer Biotech Company (Hangzhou, China).

### Data analyses

The MS/MS data of crotonylated peptides were processed through MaxQuant with the integrated Andromeda search engine (v.1.5.2.8) [20]. The tandem mass spectra were searched against the transcriptome of the leaves of *P. ternata* (sequenced by Micrometer Biotech Company). The parameters in MaxQuant were set according to Guo et al. (2020) [23].

The Gene Ontology (GO) annotation proteome was derived from the UniProt-GOA database (http://www.ebi.ac.uk/GOA/). The domains of proteins were identified by InterProScan (a sequence analysis application) based on the protein sequence alignment method, and the InterPro domain database was used. The Kyoto Encyclopedia of Genes and Genomes (KEGG) and WoLF PSORT databases were used to annotate protein pathways and subcellular localization respectively. Motif analysis of lysine acetylation sites was performed by MoMo (motif-x algorithm) software. Enrichment-based clustering analysis was performed with the R-package following a previous study [24].

## Supplementary Information

**Additional file 1: Fig. S1.** The flow chart of lysine crotonylation analysis (a), Distribution of Kcr-modified proteins based on the number of crotonylation sites in a protein (b). The analysis was performed based on 2106 crotonylated sites matched on 1006 proteins overlapping in three independent tests.

**Additional file 2: Supplementary Table S1. **Crotonylated sites of proteins in the leaves of *P. ternata*.

**Additional file 3: Supplementary Table S2. **Functional classification of crotonylated proteins in the leaves of *P. ternata*.

**Additional file 4: Supplementary Table S3. **GO enrichment analysis of crotonylated proteins in the leaves of *P. ternata*.

**Additional file 5: Supplementary Table S4. **KEGG pathway analysis of crotonylated proteins in the leaves of *P. ternata*.

**Additional file 6: Supplementary Table S5. **Protein domain enrichment analysis of crotonylated proteins in the leaves of *P. ternata*.

**Additional file 7: Supplementary Table S6. **Annotation of the total proteins in the leaves of *P. ternata*.

## Data Availability

The datasets generated during the current study have been deposited to the Proteome Xchange Consortium via the PRIDE partner repository with the dataset identifier PXD033142 (https://www.ebi.ac.uk/pride/archive). All data and materials used during the current study are available from the corresponding author upon reasonable request.

## Abbreviations

GO: Gene Ontology
PTMs: Posttranslational modifications
PAL: L-Phe ammonia-lyase
CA: Trans-cinnamic acid
PDC: Pyruvate decarboxylase
AHAS: Acetolactate synthase
ABA: Abscisic acid
GA: Gibberellic acid
SNX1: SORTING NEXIN 1
VPS: Vacuolar protein sorting
PP2C: Type-2C protein phosphatase
SnRKs: SNF1-related protein kinases
HSPs: Heat shock proteins
FBPase: Fructose-1,6-bisphosphatase
